# Risk of pneumonia in patients with gastroesophageal reflux disease: A population-based cohort study

**DOI:** 10.1371/journal.pone.0183808

**Published:** 2017-08-24

**Authors:** Wan-Tseng Hsu, Chih-Cheng Lai, Ya-Hui Wang, Ping-Huei Tseng, Kun Wang, Cheng-Yi Wang, Likwang Chen

**Affiliations:** 1 School of Pharmacy, National Taiwan University, Taipei, Taiwan; 2 Department of Intensive Care Medicine, Chi Mei Medical Center, Liouying, Tainan, Taiwan; 3 Medical Research Center, Cardinal Tien Hospital and School of Medicine, College of Medicine, Fu Jen Catholic University, New Taipei City, Taiwan; 4 Department of Internal Medicine, College of Medicine, National Taiwan University Hospital, Taipei, Taiwan; 5 Department of Internal Medicine, Cardinal Tien Hospital and School of Medicine, College of Medicine, Fu Jen Catholic University, New Taipei City, Taiwan; 6 Institute of Population Health Sciences, National Health Research Institutes, Zhunan, Miaoli County, Taiwan; Mathematical Institute, HUNGARY

## Abstract

**Purpose:**

The prevalence of gastroesophagel reflux disease (GERD) has steadily increased. However, the association between GERD itself and the risk of pneumonia remains unclear. This study aimed to investigate the association between GERD and long-term risk of pneumonia and to identify the major risk factors for pneumonia in GERD patients.

**Methods:**

Using the Taiwan National Health Insurance Research Database, we identified patients who were newly diagnosed with GERD and treated with proton pump inhibitors (PPIs) from January 1, 2004 through December 31, 2010. Two groups comprising 15,715 GERD cases and 15,715 non-GERD matched controls were generated using propensity score matching, thereby making the differences in basic demographics, concomitant medication use, and comorbidities between the two groups inconsiderable.

**Results:**

Cumulative incidence of pneumonia was significantly higher in the patients with GERD than that in the non-GERD matched controls, with an adjusted HR of 1.48 (95% confidence interval [CI] = 1.31–1.67; *P* < 0.001) within 6-year follow-ups. Multivariate stratified analyses revealed similar results in many subgroups, with a highest risk in individuals younger than 40 years of age (HR = 2.17, 95% CI = 1.48–3.19). Crucially, patients with GERD using PPIs longer than 4 months were at a significantly increased risk of pneumonia than those who did not use PPIs or took PPIs less than 4 months.

**Conclusions:**

GERD was significantly associated with long-term risk of pneumonia, especially in GERD with PPI use longer than 4 months or in the young population. Further prospective longitudinal studies should be conducted for validation and implementing clinical practice guidelines.

## Introduction

Gastroesophageal reflux disease (GERD) is a common disorder with prevalence ranging from 10% to 28% in Western countries and from 2.5% to 7.8% in Asia [1]. GERD is associated with pathologic reflux and has extraesophageal manifestations. Respiratory disorders, including recurrent pneumonia, asthma, chronic obstructive pulmonary disease (COPD), and idiopathic pulmonary fibrosis, result in bothersome clinical symptoms and complications [2]. Although of uncertain etiology, epithelium damage and inflammation caused by reflux materials are proposed as pivotal causes of GERD-related pulmonary diseases [3].

Proton pump inhibitors (PPIs) are indicated for a variety of gastric acid-related conditions, including GERD, peptic ulcer disease, *Helicobacter Pylori* infections, and other hypersecretory conditions. Although PPIs are generally well-tolerated and extremely effective for acid suppression, their use does not come without adverse effects. The use of potent PPIs has been proposed to cause an increase in gastric pH and is in connection with community-acquired respiratory tract infection, definitely pneumonia [4–7].

Taiwan’s National Health Insurance (NHI) covers over 99% of the population of Taiwan. The Bureau of NHI, Taiwan, and the National Health Research Institutes, Taiwan, maintain a NHI Research database (NHIRD) and give permission to access data for research purposes. Since the public release of Taiwan’s NHIRD in 2000, it has become one of the most productive large-scale administrative health care databases in the world and has been applied extensively in numerous studies. Studies utilizing Taiwan’s NHIRD were still not fully conclusive regarding PPI’s long-term harm. Abundant with the clinical diagnosis and various data regarding drug prescriptions of almost 23 million residents in Taiwan, NHIRD studies could inform us of the associations between clinical outcomes and PPI therapy. It is imperative to identify the critical risk factors for PPI use within a vulnerable population. Informative findings derived from the NHIRD would help clinicians reduce uncertainties about the incidence of morbidity and mortality as well as balance the risks and benefits of PPIs for high risk patients.

We hypothesized that GERD patients with concomitant PPI use have a higher risk of pneumonia events than individuals without GERD. The purpose of this study aimed to more definitively investigate the association between GERD with PPI use and subsequent long-term risk of pneumonia by conducting the population-based cohort study based on propensity score matching analysis for an adequate follow-up period.

## Methods

### Ethical consideration

In this study, ethics approval was approved by the Institutional Review Board (IRB) of Cardinal Tien Hospital.

### Data source

We acquired a subset of the NHIRD with one million random subjects, containing nearly 5% of all subjects enrolled in the NHI program. The differences in age, sex, or health care costs between the sample group and all enrollees were negligible (data not shown). The database comprises medical claims information pertinent to ambulatory care, inpatient care, dental services, and prescription drugs as well as insurance data from all beneficiaries. Most importantly, it covers nearly all clinical activities, including date, expenditures, and diagnosis related to both inpatient and outpatient procedures; prescription details; examinations; and operations. The International Classification of Diseases, Ninth Revision, Clinical Modification (ICD-9-CM) coding system, was incorporated into the data and was used in this study.

### Identification of the GERD group and the non-GERD group

**Fig 1:**
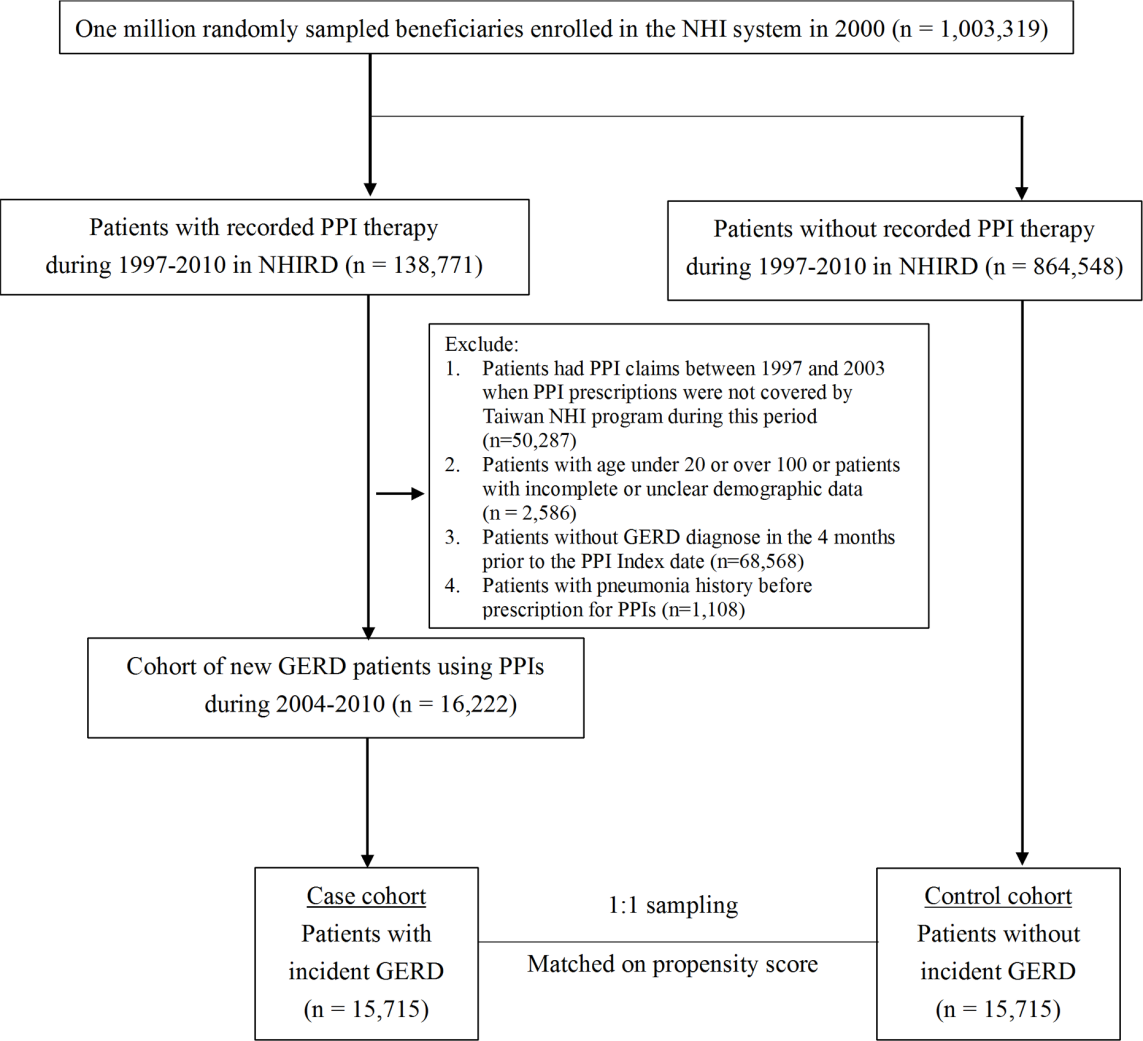
Flow chart of patient inclusion.

GERD patients was diagnosed by esophago-gastroscopy and then prescribed with PPIs. PPIs included omeprazole, pantoprazole, lansoprazole, rabeprazole, and esomeprazole. The index date was defined as the initial date of PPI prescription. In this retrospective cohort study, we defined a cohort of 16,222 adult subjects (aged between 20 and 100 years) who were newly diagnosed as having GERD (ICD-9-CM codes: 530.85, 530.11, or 530.81) and who subsequently prescribed PPIs for treatment of the disease during 2004–2010. Eligibility criteria were as follows: patient aged between 20 and 100 years with complete demographic data; no antecedent PPI prescription within 4 months; and no pneumonia history before prescription for PPIs. Next, all beneficiaries who have GERD were extracted using ICD-9-CM coding (530.85, 530.11, and 530.81). GERD patients were identified not only with ICD-9-CM codes and also with procedure codes of endoscopy. Patients who no more have GERD diagnosis after receiving endoscopy were excluded. Patients were also excluded if they had previous history of peptic ulcer disease. We further sampled the non-GERD group and conducted a propensity-score matched analysis to minimize the imbalance of baseline characteristics between the GERD and the non-GERD group (Fig 1).

### Baseline variables

We collected demographic profiles and clinical characteristics of study subjects, including age, sex, monthly income (NT$ < 19,100, NT$ = 19,100–41,999, and NT$ ≥ 42,000), comorbidities, and concomitant medication. Comorbidities were defined according to ICD-9-CM and procedure codes within 1 year before patients’ index admissions. We used a relatively conservative criterion to define comorbidities as previously described: coding any morbidity requires at least one admission or three out-patient clinic visits for disease treatment during the past year before index admission [8]. As reported previously, the most typical prescription for PPIs was 4 months in duration [9]. Because of this, we used a follow-up period of 4 months after the first PPI prescription date for the exposed cohort in the present study. We also collected prescription details, including dispensing date, quantity, dose, and duration of drug supply.

### Definition of outcome

We followed up each enrollee until December 31, 2010 to observe the development of de novo pneumonia. The follow-up duration was calculated from the diagnosis of GERD to the date of first pneumonia coded.

### Statistical analysis

We utilized SAS, version 9.4 (SAS Institute) to analyze all data. Continuous variables were reported as mean ± standard deviations (SD), and compared with Student’s t test; Categorical variables were expressed as numbers or percentages, and examined by chi-square test.

The incidence rates of pneumonia in the two groups were calculated using Poisson distribution. To assess the risk of developing pneumonia, we conducted a multivariate survival analysis using Cox regression models to examine the hazard ratio (HR) of the two cohorts for pneumonia. Covariate adjustment using the propensity score was then applied to minimize the effects of any residual confounding [10].

Kaplan-Meier method was also used to estimate the cumulative incidence rate of pneumonia for patients with or without GERD. Comparison of cumulative incidence curves between groups was evaluated by log-rank test. Two-sided *P* values < 0.05 were considered statistically significant.

## Results

As mentioned in methods, there are 15,715 newly diagnosed GERD patients and 15,715 control (non-GERD) patients. Baseline characteristics and comorbid medical disorders are listed in Table 1 and S1 Table. Through propensity score matching, the data is balanced between GERD and control cohort. Study subjects were almost equal in gender (51% men), and the mean age was 48.3 years (SD = 16.0 years). The percentage distribution of monthly income did not differ significantly between these two cohorts. The five most common comorbidities identified in the study population were hypertension, followed by diabetes, COPD, moderate to severe liver disease, and cerebrovascular disease. Compared to the controls, GERD patients also displayed comparable rates of comorbidities and similar medication profiles (Table 1).

**Table 1:** Demographic characteristics and comorbidity in study population.

Variables	GERD	Non-GERD	*p-value*
	(n = 15,715)	(n = 15,715)	
Age, mean ± SD (year)	48.32 ± 15.65	48.32 ± 16.52	0.9888
Male gender, n (%)	8,112 (51.62)	7,973 (50.73)	0.1168
Index date ^a^			0.9984
Monthly income, n (%)			
Dependent	2,899 (18.45)	2,938 (18.70)	0.5857
< NT$19,100	3,281 (20.88)	3,252 (20.69)	
NT$19,100–41,999	6,585 (41.90)	6,657 (42.36)	
≧ NT$42,000	2,950 (18.77)	2,868 (18.25)	
Baseline Comorbidities			
Myocardial infarction, n (%)	44 (0.28)	41 (0.26)	0.7445
Congestive heart failure, n (%)	159 (1.01)	155 (0.99)	0.8205
Peripheral vascular disease, n (%)	54 (0.34)	50 (0.32)	0.6944
Cerebrovascular disease, n (%)	508 (3.23)	502 (3.19)	0.8478
Dementia, n (%)	80 (0.51)	82 (0.52)	0.8748
Tuberculosis, n (%)	27 (0.17)	24 (0.15)	0.6742
Chronic obstructive pulmonary disease, n (%)	683 (4.35)	642 (4.09)	0.2498
Bronchiectasis, n (%)	24 (0.15)	19 (0.12)	0.4455
Hemiplegia or paraplegia, n (%)	38 (0.24)	33 (0.21)	0.5525
Chronic kidney disease, n (%)	219 (1.39)	232 (1.48)	0.5375
Moderate or severe liver disease, n (%)	598 (3.81)	575 (3.66)	0.4937
Tumor, n (%)	378 (2.41)	393 (2.50)	0.5844
Hypertension, n (%)	2,942 (18.72)	2,911 (18.52)	0.6533
Diabetes, n (%)	1,166 (7.42)	1,185 (7.54)	0.6837
Other Medication			
Histamine-2 receptor antagonists, n (%)	6,102 (38.83)	6,242 (39.72)	0.1059
Aspirin, n (%)	773 (4.92)	790 (5.03)	0.6591
Clopidogrel, n (%)	166 (1.06)	152 (0.97)	0.4301
Ticlopidine, n (%)	67 (0.43)	71 (0.45)	0.7329
Statins, n (%)	1,152 (7.33)	1,131 (7.20)	0.6481
Nonsteroidal anti-inflammatory drugs, n (%)	11,861 (75.48)	11,944 (76.00)	0.2748
Insulin, n (%)	310 (1.97)	283 (1.80)	0.2630

GERD = gastroesophageal reflux disease; NT$ = New Taiwan dollar

^a ^The complete profile of the index date is listed in S1 Table in the supporting information.

Overall, a total of 650 cases had pneumonia among GERD patients during the follow-up of 41,765 person-years, and the rate of pneumonia was 156 per 10,000 person-years. In contrast, a total of 444 cases had pneumonia among patients without GERD during the follow-up of 42,390 person-years, and the rate of pneumonia was 105 per 10,000 person-years. After adjusting all covariates, the overall incidence of pneumonia remained significantly higher in GERD patients than in control patients (adjusted HR = 1.48; 95% CI = 1.31–1.67; Table 2). Table 2 also demonstrated that patients with longer PPI use would have higher risk of pneumonia. As compared to patients without PPI use, the incidence of pneumonia was higher in patients with PPI use longer than 4 months (adjusted HR = 1.93; 95% CI = 1.64–2.28) than in patients with PPI use less than 4 months (adjusted HR = 1.33; 95% CI = 1.17–1.52), and both were significantly higher than the control.

**Table 2:** Cox proportional hazard ratio (HR) of pneumonia between GERD and non-GERD cohorts or among patients who used proton pump inhibitors (PPIs) longer than 4 months, less than 4 months and those who did not use PPIs.

	Pneumonia events	Observed person-years	IR^a^	Crude HR	Adjusted HR^b^
				[95% CI]	[95% CI]
GERD diagnosis					
Non-GERD	444	42390.69	105	reference	reference
GERD	650	41765.03	156	1.48 [1.32, 1.67]	1.48 [1.31, 1.67]
Duration of PPI use					
Non-GERD (without PPI use)	444	42390.69	105	reference	reference
PPI use < 4 months	438	31535.34	139	1.32 [1.16, 1.51]	1.33 [1.17, 1.52]
PPI use≧4 months	212	10229.69	207	1.98 [1.68, 2.33]	1.93 [1.64, 2.28]

CI = confidence interval; HR = hazard ratio; IR = incidence rate

^a^Incidence rate expressed as per 10,000 person-years

^b^Adjusted for propensity score

**Fig 2:**
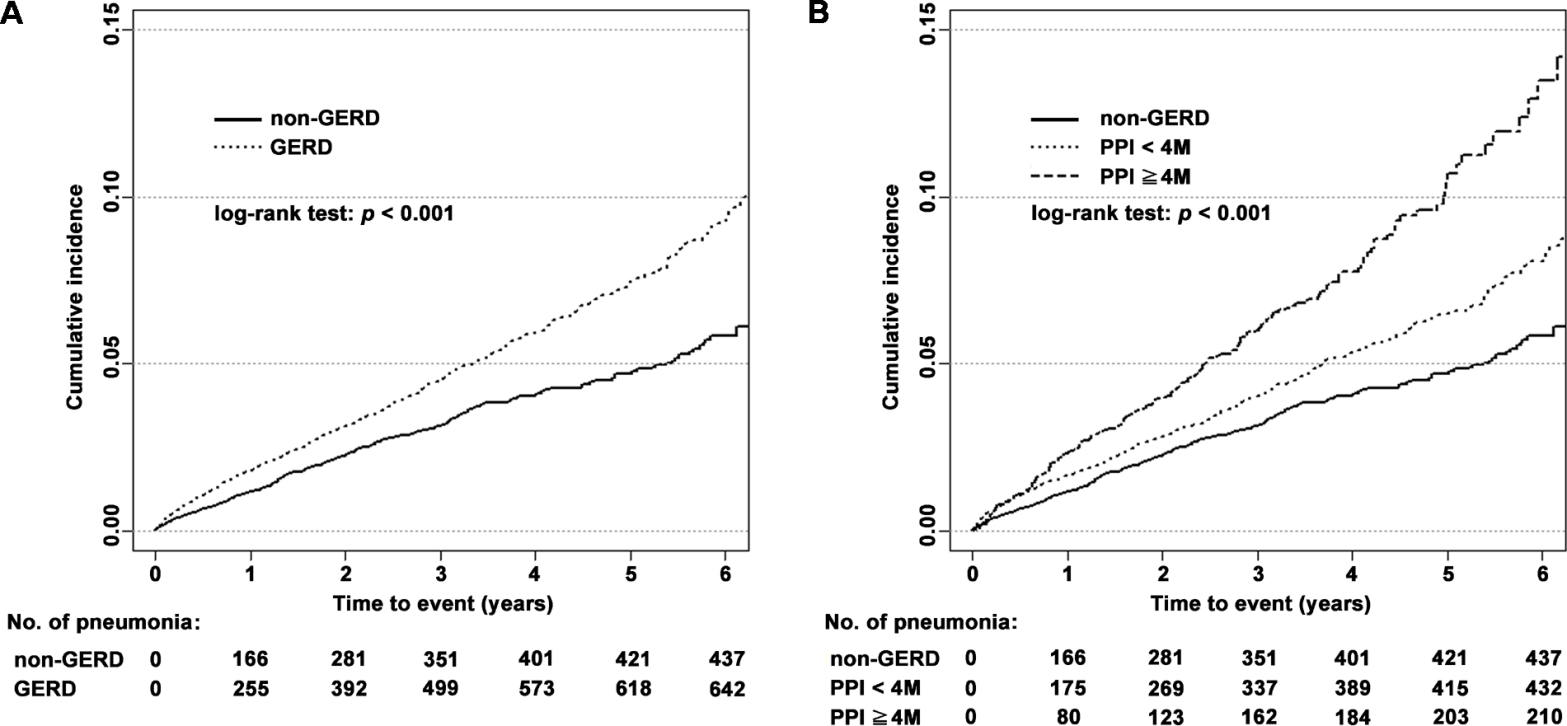
Kaplan–Meier curves of the occurrence of pneumonia for different groups. (A) GERD vs. non-GERD; (B) PPI use < 4 months (4M) vs. PPI use ≧ 4 months (4M) vs. without PPI use (non-GERD).

Fig 2A displayed that the cumulative incidence of pneumonia is higher in GERD patients than the comparison cohort (Log rank test, *P* < 0.001). In a secondary analysis, we stratified current PPI use into prescriptions less or longer than 4 months (Table 2). Compared with nonuse, longer duration of PPI use was associated with a higher pneumonia risk (adjusted HR = 1.98, 95% CI = 1.68–2.33, Table 2; Log rank test, *P* < 0.001, Fig 2B). Subgroup analyses according to sex, age, income, and the five most common comorbidities identified in the study population were also performed (Table 3). Both male and female patients had higher risk of pneumonia if they have GERD, but the association was more prominent in female (Table 3). In age group analysis, no matter the age of patients were 20–39, 40–59 or older than 60, GERD patients have higher pneumonia incidence (Table 3). In addition, GERD had the strongest association with pneumonia in the younger patients, defined as younger than 40 years old (adjusted HR = 2.17; 95% CI = 1.48–3.19; Table 3). Moreover, GERD patients have higher pneumonia incidence regardless of their income level and the presence or absence of comorbid hypertension, diabetes, COPD, liver disease, and cerebrovascular disease (Table 3).

**Table 3:** Comparison of incidence rate and hazard ratio of pneumonia stratified by sex, age and comorbidities between GERD and non-GERD cohorts.

		GERD			non-GERD			Adjusted HR^b^ [95%CI]	
		Events	Person-years	IR^a^	Events	Person-years	IR^a^		
Sex									
	M	391	8,035,473	49	275	7,823,933	35	1.39 [1.19,1.63]	
	F	259	7,219,206	36	169	7,659,265	22	1.61 [1.33,1.96]	
Age group									
	20–39	78	5,161,024	15	39	5,620,574	7	2.17 [1.48,3.19]	
	40–59	156	6,806,236	23	93	6,392,268	15	1.57 [1.21,2.03]	
	≧60	416	3,287,419	127	312	3,470,356	90	1.42 [1.22,1.64]	
Income									
	Dependent	202	2,744,477	742	136	2,795,659	49	1.52 [1.22,1.89]	
	< NT$19,100	193	3,222,682	60	130	3,211,941	40	1.48 [1.19,1.85]	
	NT$19,100–41,999	228	6,468,152	35	160	6,554,454	24	1.45 [1.19,1.78]	
	≧NT$42,000	27	2,819,368	10	18	2,921,144	6	1.52 [0.84,2.77]	
Hypertension									
	Yes	254	2,708,928	94	193	2,588,059	75	1.26 [1.05,1.53]	
	No	396	12,545,751	32	251	12,895,139	19	1.61 [1.37,1.89]	
Diabetes									
	Yes	128	1,042,432	123	105	1,043,668	100	1.23 [0.95,1.60]	
	No	522	14,212,247	37	339	14,439,530	23	1.56 [1.36,1.79]	
COPD									
	Yes	108	710,594	152	75	621,518	121	1.30 [0.96,1.74]	
	No	542	14,544,085	37	369	14,861,680	25	1.50 [1.31,1.71]	
Moderate or severe liver disease									
	Yes	32	611,590	52	39	527,290	74	0.73 [0.46,1.17]	
	No	618	14,643,089	42	405	14,955,908	27	1.56 [1.37,1.76]	
Cerebrovascular disease									
	Yes	105	464,473	226	76	445,363	171	1.31 [0.97,1.76]	
	No	545	14,790,206	37	368	15,037,835	24	1.50 [1.31,1.71]	

COPD = Chronic obstructive pulmonary disease; IR = incidence rate; NT$ = New Taiwan dollar

^a^Incidence rate expressed as per 1,000,000 person-years

^b^Adjusted for propensity score

## Discussion

To the best of our knowledge, this is the first population-based cohort study utilizing Taiwan’s NHIRD to present that GERD patients exhibited a 48% higher risk of developing pneumonia than the non-GERD individuals. In addition, the sensitivity test demonstrated GERD patients carried a higher risk of pneumonia irrespective of age, gender, income and underlying disease. In particular, the risk is significantly increased in the subgroup of female gender (adjusted HR 1.61, 95% CI = 1.33–1.96) and younger population (adjusted HR 2.17, 95% CI = 1.48–3.19). Moreover, the association between GERD and pneumonia still remains significant after discontinuous use of PPI, and thus hints that without PPI use, GERD itself can increase the risk of pneumonia. All of these findings should indicate that GERD is significantly in relation to the risk of pneumonia.

The key theory that has shaped the association between PPIs and pneumonia is the increase in gastric pH with acid-suppressive therapy, which may create and maintain a milieu in the oral cavity for intestinal pathogens to colonize [11, 12]. However, most studies were based on insufficient risk-adjusted design whereby the relatively modest increased risk could be attributed to residual confounding. Other lines of evidence were limited in a small-scale or a single-center study. Furthermore, previous studies found that the association was weakest among current recipients who had been treated with PPIs for the longest duration. This opposite temporal trend is inconsistent with a positive correlation between PPIs and the risk for pneumonia [4, 11, 12]. Previous studies conducted using the NHIRD have shown that patients with stroke [13] or COPD [14, 15] have non-significant risk of pneumonia. By contrast, PPI use in individuals with nontraumatic intracranial hemorrhage was at a high risk of long-term pneumonia [16]. Therefore, the association between PPI use and long-term risk of pneumonia was not well established.

In this study, the duration of PPI exposure was positively associated with increased risk of pneumonia in the GERD cohort, indicating that the risk of pneumonia was more noticeable, or lasting, among current recipients who had been taking PPIs for much longer periods. By contrast, several lines of evidence have demonstrated that increased risk for pneumonia was only associated with recent PPI use [17] or short-duration PPI therapy (< 30 days) [12], whereas longer periods of current PPI use were not associated with an increased risk [17]. The disparity between the findings of the present analysis and those of previous studies might be attributed to that patients with comorbid conditions or poor health status were more likely to take PPIs for longer time [17]. However, these patients are already at intrinsically higher risk for pneumonia owing to more compromised immunity and/or more frequent exposure to nosocomial pathogen. Therefore, the absolute effect of PPI use on increased risk of pneumonia might be mitigated.

The mechanism behind development of pneumonia in GERD patients under PPI treatment may be multifactorial. First, GERD patients are prone to have occult microaspiration, which pathologically connects GERD and lung disease [2]. Second, chronic PPI use can compromise the stomach’s acid-secretion capability. Acid-labile pathogen might be colonized and subsequently aspirated as this physiologic defense in the stomach has started to deteriorate [18]. Third, PPIs may reduce the acidity of the upper aerodigestive tract through their inhibition of extra-gastric H^+^/K^+^-ATPase enzymes. Consequently, bacterial colonization of the larynx, esophagus and lungs ensues [18]. Forth, in vitro studies also suggest that PPIs may suppress the activity of neutrophils, cytotoxic T-lymphocytes and natural killer cells, which might increase susceptibility to pulmonary infection and pneumonia [19–21].

Based on our findings, we proposed that PPI treatment can potentiate GERD’s complication on pneumonia. This finding has a significant clinical implication. Severer cases of GERD are more dependent on PPI treatment than milder one; often, GERD patients at this stage will have already undergone prolonged duration of PPI therapy, making them more prone to suffering incidental pneumonia. A vicious cycle ensues that patients with severer GERD have a greater risk of microaspiration, which suppresses airway immunity, and consequently more respiratory epithelium damage. Although the use of acid-suppressive PPIs may reduce repetitive acid aspiration, the epithelial damage actually occurs before the advance of GERD. Clinicians should be more carefully considered persistent acid suppression with PPIs in this scenario.

Collection of records of medical visits and treatment in NHIRD is comprehensive. The database covers nearly all clinical activities, including date, expenditures, and diagnosis related to both outpatient and inpatient procedures; prescription details; examinations; and operations. However, it raised the question whether the diagnosis was accurate when information including results of biochemical measures, imaging examinations, clinical risk score, and lifestyle were inaccessible. In this study, the diagnosis of GERD was confirmed by ICD coding, endoscopy, and PPI prescriptions to ensure data validity. Further study comparing the NHIRD data to the medical charts of the identified GERD cases from a selected medical center in Taiwan may help to evaluate its accuracy and reliability.

This study had several strengths that supported the validity of our findings. First, we performed a propensity score matching approach to create a matched control cohort that balanced the distribution patterns of many covariates, which may have affected the risk of pneumonia, thus minimizing potential selection bias. Second, because Taiwanese citizens are covered by a single NHI system, we could consider extensive medical risks and track a comprehensive population through their follow-up records. Third, to improve the accuracy of GERD diagnosis, we did not use ICD codes as the only identification methods. In this study, all of GERD must be identified by the confirmation of endoscopic examination. Last, a large sample size enabled us to conduct various stratified analyses to investigate the association between GERD and pneumonia in many subgroups. This study also had certain limitations because of its retrospective character. Reviewing the NHIRD database to identify GERD patients and occurrence of pneumonia without confirmation by laboratory tests and hospitalization course may have resulted in the recording of artificial events. Moreover, information on the patients’ compliance of PPI prescription was not included. Hence, the clinical significance of the detected risk of pneumonia should be established in prospective studies which enrolled GERD patients to monitor drug compliance. In addition, this study excluded patients who suffered pneumonia before PPI index date. Therefore, the risk of recurrent pneumonia in this population was not identified. Finally, the findings mining from the NHIRD reflect the genetics, disease patterns, health seeking behavior, and social and medical practices of Taiwanese. The fact that these factors can vary among different countries and ethnicities would limit the generalizability of these results.

## Conclusions

From Taiwan’s NHIRD, we successfully identified the significant provoking of pneumonia arising from GERD treated with PPIs based on propensity score matching analysis and an appropriate follow-up period. Our findings implicate that clinicians should be aware of increased risk of pneumonia for prescribing PPIs to younger GERD patients, who are usually without other comorbidity, and to severer GERD cases, whose demand for duration of PPI therapy may be longer. These results further support the promise of population-based cohort study in Taiwan as a platform for delineating disease complication, investigating medication adverse effects, and developing clinical practice guideline as a whole.

## Supporting information

S1 Table The index date of GERD patients and non-GERD controls. (DOCX)
