# Supplementary material for: Risk of pneumonia in patients with gastroesophageal reflux disease: A population-based cohort study
Source: PLoS One. 2017 Aug 24;12(8):e0183808. doi: 10.1371/journal.pone.0183808 (PMC5570340; doi:10.1371/journal.pone.0183808)
Supplement: S1 Table — (DOCX) [file pone.0183808.s001.docx]

**Supporting information**

**STable1 The index date of GERD patients and non-GERD controls**

| Index date  (year, month; YYYY-MM) | Non-GERD  (n = 15715) | GERD  (n = 15715) | Total  (n = 31430) |
| --- | --- | --- | --- |
| 2004-01, n (%) | 28 (0.18) | 46 (0.29) | 74 |
| 2004-02, n (%) | 50 (0.32) | 59 (0.38) | 109 |
| 2004-03, n (%) | 75 (0.48) | 76 (0.48) | 151 |
| 2004-04, n (%) | 89 (0.57) | 89 (0.57) | 178 |
| 2004-05, n (%) | 87 (0.55) | 97 (0.62) | 184 |
| 2004-06, n (%) | 78 (0.50) | 79 (0.50) | 157 |
| 2004-07, n (%) | 60 (0.38) | 78 (0.50) | 138 |
| 2004-08, n (%) | 96 (0.61) | 90 (0.57) | 186 |
| 2004-09, n (%) | 65 (0.41) | 83 (0.53) | 148 |
| 2004-10, n (%) | 101 (0.64) | 96 (0.61) | 197 |
| 2004-11, n (%) | 94 (0.60) | 89 (0.57) | 183 |
| 2004-12, n (%) | 110 (0.70) | 99 (0.63) | 209 |
| 2005-01, n (%) | 120 (0.76) | 116 (0.74) | 236 |
| 2005-02, n (%) | 67 (0.43) | 76 (0.48) | 143 |
| 2005-03, n (%) | 101 (0.64) | 93 (0.59) | 194 |
| 2005-04, n (%) | 99 (0.63) | 89 (0.57) | 188 |
| 2005-05, n (%) | 89 (0.57) | 92 (0.59) | 181 |
| 2005-06, n (%) | 101 (0.64) | 101 (0.64) | 202 |
| 2005-07, n (%) | 109 (0.69) | 113 (0.72) | 222 |
| 2005-08, n (%) | 116 (0.74) | 114 (0.73) | 230 |
| 2005-09, n (%) | 120 (0.76) | 122 (0.78) | 242 |
| 2005-10, n (%) | 138 (0.88) | 144 (0.92) | 282 |
| 2005-11, n (%) | 133 (0.85) | 126 (0.80) | 259 |
| 2005-12, n (%) | 154 (0.98) | 146 (0.93) | 300 |
| 2006-01, n (%) | 109 (0.69) | 128 (0.81) | 237 |
| 2006-02, n (%) | 144 (0.92) | 142 (0.90) | 286 |
| 2006-03, n (%) | 177 (1.13) | 178 (1.13) | 355 |
| 2006-04, n (%) | 188 (1.20) | 152 (0.97) | 340 |
| 2006-05, n (%) | 186 (1.18) | 186 (1.18) | 372 |
| 2006-06, n (%) | 136 (0.87) | 142 (0.90) | 278 |
| 2006-07, n (%) | 161 (1.02) | 145 (0.92) | 306 |
| 2006-08, n (%) | 159 (1.01) | 164 (1.04) | 323 |
| 2006-09, n (%) | 163 (1.04) | 186 (1.18) | 349 |
| 2006-10, n (%) | 159 (1.01) | 153 (0.97) | 312 |
| 2006-11, n (%) | 199 (1.27) | 178 (1.13) | 377 |
| 2006-12, n (%) | 198 (1.26) | 179 (1.14) | 377 |
| 2007-01, n (%) | 221 (1.41) | 210 (1.34) | 431 |
| 2007-02, n (%) | 132 (0.84) | 146 (0.93) | 278 |
| 2007-03, n (%) | 240 (1.53) | 233 (1.48) | 473 |
| 2007-04, n (%) | 224 (1.43) | 218 (1.39) | 442 |
| 2007-05, n (%) | 225 (1.43) | 220 (1.40) | 445 |
| 2007-06, n (%) | 211 (1.34) | 203 (1.29) | 414 |
| 2007-07, n (%) | 204 (1.30) | 231 (1.47) | 435 |
| 2007-08, n (%) | 195 (1.24) | 195 (1.24) | 390 |
| 2007-09, n (%) | 184 (1.17) | 196 (1.25) | 380 |
| 2007-10, n (%) | 267 (1.70) | 240 (1.53) | 507 |
| 2007-11, n (%) | 216 (1.37) | 224 (1.43) | 440 |
| 2007-12, n (%) | 239 (1.52) | 223 (1.42) | 462 |
| 2008-01, n (%) | 205 (1.30) | 243 (1.55) | 448 |
| 2008-02, n (%) | 243 (1.55) | 217 (1.38) | 460 |
| 2008-03, n (%) | 238 (1.51) | 244 (1.55) | 482 |
| 2008-04, n (%) | 238 (1.51) | 237 (1.51) | 475 |
| 2008-05, n (%) | 251 (1.60) | 227 (1.44) | 478 |
| 2008-06, n (%) | 175 (1.11) | 181 (1.15) | 356 |
| 2008-07, n (%) | 174 (1.11) | 171 (1.09) | 345 |
| 2008-08, n (%) | 214 (1.36) | 225 (1.43) | 439 |
| 2008-09, n (%) | 206 (1.31) | 195 (1.24) | 401 |
| 2008-10, n (%) | 239 (1.52) | 230 (1.46) | 469 |
| 2008-11, n (%) | 239 (1.52) | 239 (1.52) | 478 |
| 2008-12, n (%) | 187 (1.19) | 221 (1.41) | 408 |
| 2009-01, n (%) | 184 (1.17) | 181 (1.15) | 365 |
| 2009-02, n (%) | 201 (1.28) | 213 (1.36) | 414 |
| 2009-03, n (%) | 291 (1.85) | 269 (1.71) | 560 |
| 2009-04, n (%) | 295 (1.88) | 288 (1.83) | 583 |
| 2009-05, n (%) | 242 (1.54) | 244 (1.55) | 486 |
| 2009-06, n (%) | 288 (1.83) | 263 (1.67) | 551 |
| 2009-07, n (%) | 288 (1.83) | 268 (1.71) | 556 |
| 2009-08, n (%) | 264 (1.68) | 260 (1.65) | 524 |
| 2009-09, n (%) | 223 (1.42) | 252 (1.60) | 475 |
| 2009-10, n (%) | 255 (1.62) | 271 (1.72) | 526 |
| 2009-11, n (%) | 281 (1.79) | 278 (1.77) | 559 |
| 2009-12, n (%) | 249 (1.58) | 247 (1.57) | 496 |
| 2010-01, n (%) | 269 (1.71) | 272 (1.73) | 541 |
| 2010-02, n (%) | 206 (1.31) | 222 (1.41) | 428 |
| 2010-03, n (%) | 304 (1.93) | 309 (1.97) | 613 |
| 2010-04, n (%) | 248 (1.58) | 270 (1.72) | 518 |
| 2010-05, n (%) | 250 (1.59) | 257 (1.64) | 507 |
| 2010-06, n (%) | 273 (1.74) | 280 (1.78) | 553 |
| 2010-07, n (%) | 296 (1.88) | 280 (1.78) | 576 |
| 2010-08, n (%) | 275 (1.75) | 267 (1.70) | 542 |
| 2010-09, n (%) | 270 (1.72) | 266 (1.69) | 536 |
| 2010-10, n (%) | 247 (1.57) | 270 (1.72) | 517 |
| 2010-11, n (%) | 275 (1.75) | 291 (1.85) | 566 |
| 2010-12, n (%) | 315 (2.00) | 282 (1.79) | 597 |
